# Rank, strain, and corruption among Chinese public officials: A general strain theory perspective

**DOI:** 10.3389/fpsyt.2022.1077544

**Published:** 2022-12-16

**Authors:** Kangqing Wang, Mengliang Dai, Yiwei Xia

**Affiliations:** ^1^School of Criminal Justice, China University of Political Science and Law, Beijing, China; ^2^Faculty of Law, Macau University of Science and Technology, Taipa, Macao SAR, China; ^3^School of Law, Southwestern University of Finance and Economics, Chengdu, China

**Keywords:** rank of public officials, strain, corruption, general strain theory (GST), China

## Abstract

The problem of corruption has long been a societal focus in China. Agnew’s general strain theory (GST) offers a good explanation of the drive to engage in corruption; that is, offenders are likely to be driven by various types of strains and engage in corrupt behavior as a coping mechanism. However, whether and how an official’s rank moderates the effect of strain on corrupt behavior has yet to be explored. The current study surveyed 687 inmates from 60 prisons in China who had been convicted of corrupt behaviors. The results show that although different levels of officials experience different types of strain, nearly all types of strains are significantly and positively associated with the frequency of corrupt behavior. As for the conditional effect, officials’ ranks significantly moderate the relationship between work-related strain and the frequency of corrupt behavior; that is, work-related strain is shown to have a more significant effect on officials at the clerk level (a higher rank) than on officials at non-clerk level (a lower rank). This research is believed to further expand on the applicability of GST to corruption in non-Western societies.

## Introduction

China has experienced tremendous economic development over the past four decades, during which time the problem of corruption has been a continual societal focus in the country. Corruption involving public officials has become most prominent at the grassroots level. “Grassroots public officials” is defined as officials working in township/village administrative organizations and their subordinates or officials working in grassroots organizations (e.g., residential committees or village councils). These officials, regardless of rank, are frequently involved in corruption (1).

However, as Pasculli and Ryder (2) pointed out, the study of corruption is relatively young; furthermore, evidence of the extent and the dynamics in corrupt schemes is difficult to collect due to their secretive nature. China is no exception. Therefore, most of the relevant literature has used objective data to examine the features and causes of corruption, such as judicial judgments (1, 3, 4), government-produced yearbooks (5–9), and the media (10). Some studies have relied on fieldwork and interview data to examine Chinese corruption [e.g., (11, 12)]. Moreover, some research has used surveys to study the public’s attitude toward corruption [e.g., (13, 14)]. All in all, very little research has targeted offenders (15–17). For example, based on rational choice theory, Li and Li (15) interviewed 10 offenders and surveyed 170 offenders of duty-related crimes and found that although offenders weigh the risks and returns of committing a crime, the offenders do not completely depend upon a rational-choice model to make a decision. Thus, the rational-choice model is not sufficient to explain the causes of corruption.

Previous studies have mostly attributed the cause of corruption in China to some macro-level factors, such as top-down political system, planned economic system, unique society, and culture. However, according to Coleman’s “bathtub” model of social change (18), macro-level factors could not directly affect micro-level action, unless being transformed into micro-level factors. Thus, a plausible approach to understanding the cause of corruption is *via* general strain theory (GST), which suggests that crime is a result of certain strains, with “strains” referring to events and conditions disliked by individuals (19). GST is especially useful to explain the causes of corruption at the micro-level, because regardless of socio-economic status, individuals experience strains, such as economic strain, status-related strains, and work-related strains (20), that are more easily resolved through income-generating crimes than through aggressive acts [e.g., (17, 21)]. Thus, GST suggests previous mentioned macro-level factors could be transformed into different types of strains that could possibly trigger corrupt behavior. Indeed, GST has been found to explain corrupt behavior in China. For instance, Wang et al. (17) found that six types of strains are significantly and positively associated with the frequency of corrupt behaviors in China, namely resources strain, deviant subcultural strain, economic strain, work-related strain, political promotion strain, and *renqing* strain. These types of strains have been further sorted into four categories—namely work-related strain, work-related financial strain, personal financial strain, and status-related strain—through rigorous psychometrics tests (16).

A more recent development in GST suggests that certain individuals experiencing certain types of strain in certain circumstances are likely to engage in criminal coping (19). In other words, the effect of strains on criminal coping in the form of corrupt behaviors is conditional. In the Chinese bureaucratic system, officials of different ranks have different duties and responsibilities (22, 23); consequently, they may also differ in terms of the strains they experience in the performance of their jobs. However, studies have yet to examine whether officials’ respective levels of responsibility influence their corrupt behaviors.

Therefore, this study aims to expand on the applicability of GST to corruption in non-Western countries by comparing officials’ experienced strains and examining whether the association between strain and corrupt behavior differs according to officials’ rank in China.

## Corruption studies in the Chinese context

There is no room for doubt that corruption is widespread in China (4, 24–26). The Chinese political system is characterized by an overconcentration of power in its leaders (27). Sun (28) argued that corruption indicates economic and social problems that amount to serious crises of political legitimacy and pressures for political change. Corruption not only increases income inequality but also decreases tax revenue and, consequently, government spending on education and public health in China (6). It is widely recognized that corruption is difficult to measure due to its secretive nature and associated low detectability (3, 12, 25, 29, 30).

Nevertheless, several scholars have conducted empirical studies on corruption (3–5, 7–9, 11, 13, 17, 29, 31–33). Most of these studies have used “objective” data. To examine the causes of corruption in China, Dong and Torgler (7) used a province-level dataset from 1998 to 2007 and found that provinces with greater anti-corruption efforts, higher educational attainment, historic influence from Anglo-American religious universities, greater openness, more access to media, higher government employee wages, and a greater representation of women in the legislature are markedly less corrupt, whereas social heterogeneity, regulation, and resource abundance breed substantial corruption. Drawing on a province-level dataset, Xu et al. (9) explored the impact of religion on corruption and found that religion culture plays a positive role in restraining official’s corruption because religion influences political preference and work ethics.

Although the studies above provide some understanding of corruption, few studies have examined officials’ rank in connection with corruption, especially the ranks of grassroots public officials. The officials at the grassroots level form a large proportion of the civil service. Their ranks are associated with differing roles and responsibilities (23), leading to different types of strain and stress (34).

The effect of such a regulation can be seen, for example, in Zhang et al. (35) study of 297 frontline police officers in Luzhou city, Sichuan province. They found work stress to be one of the biggest psychological problems of such officers and that officers in leadership positions experience higher levels of stress than officers in non-leadership positions. Moreover, research has shown that corruption involves not only top officials at the ministerial level and above but also clerks, at the bottom ranks (36). Tan and Tan (37) found that the corruption of first-in-command (*yibashou*) officers at the village level accounted for 53.28% of all the corruption. Therefore, it is crucial to explore the causes of officials’ corruption at the grassroots level.

## General strain theory and empirical research

Agnew’s (38) general strain theory revises classic strain theory developed by several scholars [e.g., (39–41)] by emphasizing the mechanism from strain to criminal copping. Unlike classic strain theory, Agnew’s (42) model suggests that strain may result not only from the failure to achieve positive goals but also from the inability to escape painful situations. Therefore, Agnew (38) proposed three major types of strain: failure to achieve valued goals, removing or threatening to remove a person’s positive stimuli, and presenting or threatening to present a person with noxious or negative stimuli. And crime is one of many coping mechanisms to strain.

According to Agnew (43), “strain” refers to negative or adverse relations with others. More specifically, it occurs when others do not treat an individual as he or she would like to be treated. Strain increases the likelihood that an individual will experience negative emotions, such as disappointment, depression, and anger. Individuals with high negative emotionality are much more likely to experience aversive events and to respond to such events in an aggressive or antisocial manner (44). Strains are more likely to lead to crime when they are seen as unjust, are high in magnitude, are associated with low social control, and create some pressure or incentive to engage in criminal activities (20).

Agnew (20, 45, 46) differentiated between objective and subjective strains. “Objective strains” refers to events and conditions that are disliked by most people. “Subjective strains” refers to events and conditions that are disliked by the particular person or persons being examined. The two kinds of strains greatly differ. Agnew (20) stated that it is important to measure subjective strains and later proposed that subjective strains have a larger effect on crime than objective strains (19).

A vast number of empirical studies have directly tested GST [e.g., (43, 44, 47–53)]. These studies have generally supported the theory. Agnew and White (43) were the first to empirically examine GST. They used data from the first wave of the Rutgers Health and Human Development Project, with a total of 1,380 New Jersey adolescents aged 12, 15, and 18. The results provided support for the theory. For example, the study found that negative life events and life hassles are the most influential strain factors. Some empirical studies have been conducted in non-Western countries [e.g., (54–60)]. Most of these studies produced consistent support for GST. For example, using a sample of 1,163 adolescents from four middle schools in Shenzhen, China, Gao et al. (58) investigated how adolescent maltreatment was associated with delinquency *via* the mediating effects of social control, social learning variable, and negative emotional state in the Chinese context, based on Agnew’s revised model of GST. They found that adolescent maltreatment significantly increases a person’s level of delinquency. It also directly reduces their level of social control and increases their exposure to delinquent peers, which in turn heightens their risk of delinquency.

Studies focused on juvenile delinquency have supported GST. However, few studies have investigated the ability of GST to explain white-collar crime. There have only been a very small number of empirical studies examining GST (17, 21) to explain white-collar crime. As Agnew et al. (61) pointed out, GST is a general theory that can explain all types of crimes including white-collar crime. Strains related to white-collar crime include economic strains, status-related strains, and work-related strains. Agnew et al. (61) posited that economic strain is an important trigger of white-collar crimes committed for personal gain. Such strain is easily resolved through income-generating crimes. Using data from convicted white-collar offenders, Langton and Piquero (21) examined the ability of GST to explain white-collar offenses an found that GST is useful in predicting white-collar offenses. In a recent mixed-method study, Wang et al. (17) interviewed 23 grassroots public officials convicted of corruption, and then surveyed 687 corruption offenders from 60 prisons in China. The quantitative results showed that all six types of strain—resources strain, deviant subculture strain, economic strain, work-related strain, political promotion strain, and *renqing* strain—are significantly and positively associated with the frequency of corrupt behavior. Among which, *renqing* strain refers to the strain generated by performing an informal social obligation to another party (doing *renqing*). In the current study, grassroots public officials shoulder huge strain in maintaining the relationships with their superiors, subordinates, friends, relatives, and local residents/villages for whom they work. Especially for those officials who themselves live in the local communities, the conflict between roles and social status often stimulates the motivation for corrupt behavior (17).

The relationship between strain and criminal behavior is not constant across individuals. Agnew (19) proposed an extension of GST, suggesting that several conditional variables are useful to explain why the relationship between strain and criminal coping is not stable; he proposed a number of variables such as negative emotionality, low constraint (or low self-control), self-efficacy, social support, social control, association with criminal peers, and beliefs favorable to crime. Therefore, it is reasonable to assume that the relationship between strain and white-collar crime, such as corrupt behavior, is also moderated by other factors. Given that official ranks are distinct in terms of roles and responsibilities, rank may play a moderating role in the strain–crime nexus. However, the literature has yet to address this question.

## Materials and methods

### Data

The current study explored whether and how officials’ ranks moderate the effect of strain on corrupt behaviors. A paper-pencil survey was conducted with 687 prison inmates who had been convicted of corruption from 60 prisons in China. The participants were selected based on the type of offense they had been convicted of and their occupation. The participants were grassroots public officials convicted of corruption. For the purposes of the study, “corruption” is defined as embezzlement, bribe-taking, or misappropriation (17). The survey was conducted between January 2019 and January 2020, with average lengths between 70 and 150 min. Informed consent was obtained from each of the respondents before they completed the survey [for further details on the survey data collection, please refer to Refs. (16) and (17)].

### Measures

#### Dependent variable

The frequency of corrupt behavior before conviction was chosen as the dependent variable, which was measured by asking about the annual frequency of corrupt behavior before the corruption conviction. The answer scale ranged from 1 (Never) to 5 (Very often); a higher value indicates a higher level of corruption.

#### Independent variable

The independent variables are the types of strains, measured by the Chinese Public Official Strain Scale (CPOSS) developed by Wang et al. (16). Comprising 17 items, the CPOSS measures four types of strains that are commonly experienced by grassroots public officials in Chinese settings, namely work-related strain, work-related financial strain, personal financial strain, and status-related strain. For example, respondents will be asked: Did you feel stress to complete the indicators assigned by your original unit? The answer scale ranged from 1 (No stress at all) to 5 (Extreme stress).

#### Moderating variable

The rank of a given official’s job was measured by whether or not the official was employed at the clerk level or above. In the Chinese bureaucratic system, clerk-level officials are those serving officially budgeted, public-servant posts (*gongwuyan bianzhi*), while non-clerk officials are grassroots public employee and cadres in mass, grassroots, autonomous organizations such as village committees and neighborhood committees. Therefore, the clerk status is the most significant indicator of official hierarchy in the grassroots bureaucratic system in China. Being a clerk means not only having more power or resources to allocate but also having a higher chance of being promoted as a local leader.

#### Control variables

The demographic control variables in the quantitative analysis were sex (1 = female, 0 = male), age, ethnicity (1 = Han ethnicity, 0 = otherwise), party membership (1 = party member, 0 = otherwise), and marital status (1 = married, 0 = unmarried).

### Analytical strategy

First, a descriptive analysis of all of the variables was obtained. Second, two independent sample *t*-tests were conducted to compare the demographic variables and strains to identify differences between ranks of the convicted officials. Finally, ordered logistic regression was performed to investigate whether and how rank moderates the relationship between strains and corrupt behavior.

## Results

Table 1 presents the descriptive statistics for all of the studied variables. As shown, the surveyed respondents averaged 52.77 years old, only 5.82% were female, 85.75% were of Han ethnicity, 77.85% were party members, and 98.11% were married. As for the officials’ job rank, 74.09% of them were not at the clerk level. The respondents reported a moderate level of strain of various forms, work-related strain emerging as the most severe form, followed by status-related strain, personal financial strain, and work-related financial strain. That is, the convicted grassroots public officials reported experiencing more non-financial strain than financial strain. Finally, the average score of corruption frequency was 1.92.

**TABLE 1:** Descriptive analysis.

	*N*	Mean/%	S. D.	Min	Max
**Demographics**					
Age	678	52.77	8.75	24	80
Female	687	5.82%			
Ethnicity = Han	687	85.74%			
Party member	687	77.58%			
Married	687	98.11%			
Level of job = Clerk	687	25.91%			
**Strain**					
Overall strain	687	2.84	0.74	1	5
Work-related strain	686	3.48	0.96	1	5
Work-related financial strain	685	2.51	0.91	1	5
Personal-related financial strain	682	2.68	0.96	1	5
Status-related strain	674	3.08	1.08	1	5
**Dependent variable**					
Frequency of corruption	661	1.92	1.00	1	5

Table 2 presents a comparison of the studied variables between the clerk and non-clerk samples. Corruption-convicted clerks were significantly younger and more likely to be party members than non-clerks. Furthermore, of all of the types of strain, clerks experienced significantly more severe status-related strain and less personal-related financial strain than non-clerks. The difference in strains may be related to the content of the job and the income gap between ranks of public officials.

**TABLE 2:** Two independent sample *t*-tests between clerk and non-clerk sample.

	Not clerk (*N* = 474)	Clerk (*N* = 171)	Mean difference
**Demographics**			
Age	53.28	50.40	2.88***
Female	0.06	0.05	0.01
Ethnicity = Han	0.86	0.84	0.02
Party member	0.75	0.87	−0.12***
Married	0.98	0.98	−0.00
**Strain**			
Overall strain	2.81	2.86	−0.05
Work-related strain	3.43	3.57	−0.15
Work-related financial strain	2.48	2.54	−0.06
Personal-related financial strain	2.70	2.52	0.19*
Status-related strain	2.97	3.36	−0.39***
**Dependent variable**			
Frequency of corruption	1.89	2.05	−0.16

Standard errors in parentheses, *p < 0.05, ***p < 0.001.

Table 3 exhibits the results of the ordered logistic regression. All of the ten models estimated the effect of strain, rank, and their interactions on the frequency of delinquency. Specifically, Models 1 and 2 estimated the effect of overall strain, Models 3 and 4 estimated the effect of work-related strain, Models 5 and 6 estimated work-related financial strain, and Models 9 and 10 estimated status-related strain. Also, to evaluate the robustness of the finding, the aforementioned ten models were estimated with ordinary least square (OLS) regression (see Appendix Table 1).

**TABLE 3:** Ordered logistic regression analysis.

	Model 1	Model 2	Model 3	Model 4	Model 5	Model 6	Model 7	Model 8	Model 9	Model 10
	Overall strain	Overall strain	Work-related strain	Work-related strain	Work-related financial strain	Work-related financial strain	Personal-related financial strain	Personal-related financial strain	Status-related strain	Status-related strain
Female	−1.12**	−1.15**	−1.24***	−1.30***	−1.11**	−1.13**	−1.19**	−1.20**	−1.33***	−1.33***
	(0.37)	(0.37)	(0.37)	(0.37)	(0.38)	(0.38)	(0.37)	(0.37)	(0.37)	(0.37)
Age	−0.03**	−0.03**	−0.03**	−0.03**	−0.03**	−0.03**	−0.03**	−0.03**	−0.03**	−0.03**
	(0.01)	(0.01)	(0.01)	(0.01)	(0.01)	(0.01)	(0.01)	(0.01)	(0.01)	(0.01)
Ethnicity = Han	0.07	0.09	0.08	0.12	0.01	0.01	0.09	0.11	0.11	0.11
	(0.22)	(0.22)	(0.22)	(0.22)	(0.22)	(0.22)	(0.22)	(0.22)	(0.21)	(0.21)
Party member	0.46*	0.45*	0.50**	0.47*	0.39*	0.40*	0.52**	0.51**	0.54**	0.54**
	(0.19)	(0.19)	(0.19)	(0.19)	(0.19)	(0.19)	(0.19)	(0.19)	(0.19)	(0.19)
Married	0.24	0.21	0.18	0.15	0.38	0.35	0.22	0.20	0.26	0.25
	(0.59)	(0.59)	(0.59)	(0.59)	(0.60)	(0.60)	(0.59)	(0.58)	(0.59)	(0.59)
Job level = Clerk	0.31	1.53*	0.28	2.00**	0.31	1.02*	0.36*	0.85	0.28	0.44
	(0.17)	(0.68)	(0.17)	(0.66)	(0.17)	(0.51)	(0.17)	(0.49)	(0.17)	(0.56)
Strain	0.52***	0.64***	0.27***	0.41***	0.58***	0.66***	0.26**	0.31***	0.06	0.07
	(0.11)	(0.13)	(0.08)	(0.10)	(0.09)	(0.10)	(0.08)	(0.09)	(0.07)	(0.08)
Clerk × Strain		−0.42		−0.48**		−0.28		−0.19		−0.05
		(0.23)		(0.18)		(0.19)		(0.18)		(0.16)
Constant 1	0.42	0.74	−0.27	0.13	0.32	0.49	−0.32	−0.20	−0.84	−0.79
	(0.78)	(0.80)	(0.76)	(0.77)	(0.76)	(0.77)	(0.75)	(0.76)	(0.77)	(0.78)
Constant 2	2.04**	2.36**	1.33	1.73*	1.97**	2.15**	1.27	1.39	0.74	0.78
	(0.79)	(0.80)	(0.76)	(0.78)	(0.76)	(0.78)	(0.76)	(0.76)	(0.77)	(0.78)
Constant 3	3.16***	3.48***	2.44**	2.84***	3.12***	3.29***	2.38**	2.50**	1.84*	1.88*
	(0.79)	(0.81)	(0.77)	(0.78)	(0.77)	(0.78)	(0.76)	(0.77)	(0.77)	(0.78)
Constant 4	5.07***	5.39***	4.34***	4.75***	5.05***	5.21***	4.28***	4.41***	3.74***	3.78***
	(0.84)	(0.86)	(0.82)	(0.84)	(0.83)	(0.84)	(0.81)	(0.82)	(0.82)	(0.83)
*Pseudo R* ^2^	0.04	0.04	0.03	0.04	0.05	0.05	0.03	0.031	0.02	0.02
*AIC*	1602.88	1601.45	1616.25	1610.85	1581.76	1581.59	1617.00	1617.87	1626.85	1628.76
*BIC*	1652.05	1655.08	1665.41	1664.48	1630.93	1635.22	1666.16	1671.50	1676.01	1682.39
*N*	645	645	645	645	645	645	645	645	645	645

Standard errors in parentheses, *p < 0.05, **p < 0.01, ***p < 0.001; “Clerk × Strain” is the product of variable “job level = clerk” and “Strain”.

As shown in the table, job rank *per se* was not significantly correlated with corrupt behavior. Furthermore, with the exception of status strain, all types of strain were positively and significantly associated with corrupt behavior. More importantly, the interaction terms suggested that rank significantly moderated the effect of work-related strain on corrupt behavior (b = −0.48, *P* < 0.05), while the moderating effects of rank on the other types of strain were not statistically significant. Furthermore, the coefficient of work-related strain on corrupt behavior among non-clerks was 0.41 (*P* < 0.001), and the coefficient of work-related strain on corrupt behavior among clerks was −0.07 (0.41–0.48 = −0.07), suggesting a non-significant effect. In addition, the OLS models yielded similar findings with the ordered logistic regression shown in Table 3.

The analysis revealed that the clerk and non-clerk samples differ in status-related strain and personal financial strain. Moreover, despite the fact that nearly all types of strain were significantly associated with corrupt behavior, work-related strain may have a non-significant effect on officials in clerk-level positions and above.

## Discussion

The current study examines whether and how the hierarchy of public officials moderates the effect of strain on corrupt behaviors. The results suggest that clerks and non-clerks have different experiences of status-related strain and personal financial strain. However, despite of the differences, work-related strain is the only type that significantly differs between clerks and non-clerks.

Our findings on the differences in status-related strain and personal financial strain between clerk and non-clerk public officials validate the literature suggesting the different effects of respective roles and responsibilities of public officials of different ranks (23). Higher-ranking officials enjoy more power and manage more resources but may also shoulder greater responsibilities. Thus, they may experience higher status-related strain than non-clerk officials, which may explain why psychological problems are more prevalent in leadership positions (36). In contrast, non-clerk officials, despite experiencing less status-related strain, suffer more severe financial strain, as their salaries usually do not afford them a basic living. There is significant wage gap between clerk and non-clerk officials. As grassroots village cadres and neighborhood committee cadres belong to grassroots, self-governing, mass organizations, they have no formal salary but only meager subsidies. Thus, they generally must seek other sources of income, with corruption being an attractive option. Although clerk-level officials also experience personal financial strain, their strain is perhaps more related to the “unjust” strain described by Agnew (20) rather than true, survival-level financial strain. In sum, our findings suggest that differences in the magnitude of experienced strain may be traced to the context of the strain-generating environment. It is therefore suggested that future studies further investigate the context of strain to better understanding the mechanism of how strain ultimately develops into criminal coping.

Differences in the magnitude of strain do not necessarily mean that such strain is conditional. In the current study, the results show that official rank only moderates the effect of work-related strain on corruption. This discrepancy may be explained by the overconcentration of power in the Chinese political system (27); such a top-down power structure allows upper-level officials to use their power and resources to handle their work-related strain or even to transfer the strain to their subordinates. In contrast, lower-ranking public officials must find other ways to cope with the strain, making it more likely that they will resort to criminal coping. Thus, our results suggest that political resources may influence the effect of strain on criminal behavior (19), especially in the context of white-collar crime. This may deepen the understanding of the nature and extent of resources in the context of extended general strain theory. As Agnew (19) suggest, certain type of strain is more conductive to certain type of crime. Thus, it is useful to explore the unique types of strain that trigger corruption, which is a typical type of white-collar crime that has not been comprehensive studied. Also, Agnew suggest GST should be revised somewhat in order to best explain crime in Asian societies (62). The current study should be considered as another piece of evidence examining the generalizability of GST to Asian societies and facilitating further development of GST.

The current research has several limitations. First, the quantitative analysis may be biased by convenience sampling; despite the size of the sample, the findings may have a limited generalizability to a larger population. Second, the clerk and non-clerk samples in the interviews are unequal in size, which may lead to results that are less representative of clerk-level public officials. Despite these limitations, the study is among the first to explore rank, strain, and corruption crime in Chinese grassroots public officials with a large sample. As such, it may shed light on the drivers of white-collar crime.

## Data availability statement

The datasets presented in this article are not readily available because the study involved prison subjects. Requests to access the dataset can be available by request and are subject to the requirement of the prisons. Requests to access the datasets should be directed to KW.

## Ethics statement

The studies involving human participants were reviewed and approved by Ethics Committee, School of Law, Southwestern University of Finance and Economics. The patients/participants provided their written informed consent to participate in this study. Written informed consent was obtained from the individual(s) for the publication of any potentially identifiable images or data included in this article.

## Author contributions

YX and MD wrote the first draft of the study. KW revised the first draft and took the investigation and qualitative analysis. YX took the quantitative analysis. All authors approved the final manuscript.

## Appendix

**APPENDIX TABLE 1:** Robustness test of the main findings with ordinary least square (OLS) regression.

	Model 1	Model 2	Model 3	Model 4	Model 5	Model 6	Model 7	Model 8	Model 9	Model 10
	Overall strain	Overall strain	Work-related strain	Work-related strain	Work-related financial strain	Work-related financial strain	Personal-related financial strain	Personal-related financial strain	Status-related strain	Status-related strain
Job level = Clerk	0.10	0.51	0.08	0.80*	0.09	0.27	0.12	0.29	0.08	0.10
	(0.09)	(0.36)	(0.09)	(0.35)	(0.09)	(0.26)	(0.09)	(0.26)	(0.09)	(0.30)
Strain	0.25***	0.28***	0.12**	0.17***	0.29***	0.31***	0.11**	0.13**	0.02	0.02
	(0.05)	(0.06)	(0.04)	(0.05)	(0.04)	(0.05)	(0.04)	(0.05)	(0.04)	(0.04)
Clerk × Strain		−0.15		−0.20*		−0.07		−0.07		−0.01
		(0.12)		(0.10)		(0.10)		(0.09)		(0.09)
Control variables	Yes	Yes	Yes	Yes	Yes	Yes	Yes	Yes	Yes	Yes
Constant	1.69***	1.60***	2.05***	1.89***	1.73***	1.70***	2.08***	2.04***	2.33***	2.33***
	(0.39)	(0.40)	(0.38)	(0.39)	(0.36)	(0.37)	(0.38)	(0.38)	(0.39)	(0.39)
*R* ^2^	0.07	0.08	0.06	0.06	0.11	0.11	0.05	0.06	0.04	0.04
F	7.33	6.60	5.52	5.43	11.23	9.89	5.31	4.70	4.16	3.64
*N*	652	652	651	651	651	651	650	650	647	647

Standard errors in parentheses, **p* < 0.05, ***p* < 0.01, ****p* < 0.001.
